# A Low-Delay Lightweight Recurrent Neural Network (LLRNN) for Rotating Machinery Fault Diagnosis

**DOI:** 10.3390/s19143109

**Published:** 2019-07-14

**Authors:** Wenkai Liu, Ping Guo, Lian Ye

**Affiliations:** 1 Chongqing Key Laboratory of Software Theory and Technology, Chongqing University, Chongqing 400044, China; 2 College of Computer Science, Chongqing University, Chongqing 400044, China

**Keywords:** recurrent neural network, data-driven fault diagnosis, lightweight network, deep learning, bearing faults

## Abstract

Fault diagnosis is critical to ensuring the safety and reliable operation of rotating machinery systems. Long short-term memory networks (LSTM) have received a great deal of attention in this field. Most of the LSTM-based fault diagnosis methods have too many parameters and calculation, resulting in large memory occupancy and high calculation delay. Thus, this paper proposes a low-delay lightweight recurrent neural network (LLRNN) model for mechanical fault diagnosis, based on a special LSTM cell structure with a forget gate. The input vibration signal is segmented into several shorter sub-signals in order to shorten the length of the time sequence. Then, these sub-signals are sent into the network directly and converted into the final diagnostic results without any manual participation. Compared with some existing methods, our experiments illustrate that the proposed method has less memory space occupancy and lower computational delay while maintaining the same level of accuracy.

## 1. Introduction

Mechanical fault diagnosis—analysing data collected by sensors and predicting the health of mechanical systems—has become a research hotspot in industry [1,2]. The existing methods can be approximately divided into three categories: physics-based “white-box” models, data-driven artificial intelligence (AI) methods (“black-box”), and the combination of above two kinds, referred to as “grey-box” models. The performance of physics-based models is heavily dependent on the quality of domain knowledge about the practical mechanical systems. In reality, mechanical equipment works in complex production environments, and the collected data are seriously disturbed by a variety of noise. The models built in an ideal environment may not work in such a complex environment. A data-driven model can update parameters using real-time data [3]. This flexibility has made them become the focus of fault diagnosis research. Although white-box models are dependent on the quality of domain knowledge, this additional information could reduce the solution space and enhance the performance of black-box models. Some grey-box models have earned relatively good results in fault diagnosis. Zhou et al. [4] used neighbourhood components analysis to reduce the dimensionality of original features, then applied coupled hidden Markov model (CHMM) to bearing fault diagnosis. Jung et al. [5] exploit the multi-scale energy analysis of discrete wavelet transformation to obtain a low-dimensional feature subset of data as the input of a k-nearest neighbours ( k-NN) algorithm for bearing fault classification. Gangsar et al. [6] proposed a method for the fault diagnosis of induction motors by combining the wavelet packet transform (WPT) and support vector machine (SVM).

The traditional AI methods are also shallow models and always work on the original feature representation without creating new features during the learning process [7]. It is difficult to characterise the inherent non-linear relationship of complex mechanical systems effectively while using them. In order to further improve the diagnostic performance, deep learning has recently been applied to mechanical fault diagnosis [8]. Deep learning can automatically extract advanced features from the original data through hierarchical learning, and achieve end-to-end learning without any manual participation. Compared with the traditional AI methods, the deep learning method has lower dependence on the knowledge of feature design. At present, there are three main methods that are widely used in the field of mechanical fault diagnosis: automatic encoder (AutoEncoder), convolutional neural network (CNN), and recurrent neural network (RNN). The AutoEncoder can learn rich representation features and reduce data dimensionality, and has has received a great deal of attention in this research field. Ahmed et al. [9] proposed an unsupervised feature learning algorithm using stacked AutoEncoders to learn feature representations from compressed measurements. Lu et al. [10] presented a detailed empirical study of stacked denoising AutoEncoders with three hidden layers for fault diagnosis. Junbo et al. [11] used a digital wavelet frame and nonlinear soft threshold method to process vibration signals and used an AutoEncoder on the preprocessed signal to carry out roller bearing fault diagnosis. However, adjusting the model parameters requires a large amount of data and time, and it is difficult to judge whether the learned features are related to the target task.

CNNs can automatically select the feature of data without any artificial designs, and has been widely applied in fault diagnosis. Wen et al. [12] transformed a raw signal into a square matrix through non-overlapping cutting and normalised the value to 0–255, which was regarded as an image directly using 2D LeNet-5 for the fault prediction of gears and bearings. Liu et al. [13] cut the raw signal into a square matrix by changing the interval distance, which was used directly for fault detection in the 2D-CNN structure training. Sun et al. [14] used multi-scale information extracted by dual tree-complex wavelet transform (DT-CWT) to form a matrix and combined it with a CNN for gear fault diagnosis. Guo et al. [15] used a novel diagnosis method involving a CNN to directly classify a continuous wavelet transform scalogram (CWTS). CNNs can effectively extract local features of data and process high-dimensional data, but most CNN architectures are heavily over-parametrised and computationally complex, taking a lot of time to train the network [16].

**Figure 1:**
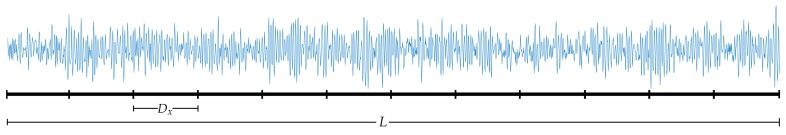
The step length ($D_{x}$) and step number (L/$D_{x}$) of a data sequence.

The initial parameter values affect the final performance of a CNN. Besides, CNNs are unable to remember historical data information. When predicting, a CNN must process historical input data repeatedly, bringing unnecessary computational consumption. The RNN is a framework for processing sequence data. It analyses the combination of historical data feature and current input data, then extracts the new feature information and makes decisions. The processing form of RNNs is more suitable for mechanical fault diagnosis. RNNs have the ability to memorise historical information, and only need to process the input data once. Therefore, they can detect the health status of mechanical systems in real time. However, the vanishing gradient problem during the back-propagation of model training hinders the performance of RNNs. This means that traditional RNNs may not capture long-term dependencies. Long short-term memory networks (LSTMs), which can extract long-term dependence features, avoid the gradient disappearance problem cleverly through the gate mechanism and have been successfully applied in various fields, including image captioning [17], speech recognition [18], and natural language processing [19]. Nevertheless, it is also difficult to train LSTMs when the data sequence is too long. LSTM-based methods also have the problem of excessive parameters and computational complexity. The LSTM structure can be further simplified to reduce the calculation time. In order to solve the above problems, this paper proposes a low-delay lightweight recurrent neural network (LLRNN) model for mechanical fault diagnosis, making the following two main contributions: (1) The design of a lightweight network structure based on a special LSTM cell with only a forget gate, reducing the parameters and calculation of the network. (2) It studies the influence of step length (i.e., the length of each sub-segment of a sequence signal, as shown in Figure 1 $D_{x}$) and step number (number of sub-segment of a sequence signal as shown in Figure 1 *L*/$D_{x}$) of the sequence data on the performance of the model, including accuracy, noise immunity, and calculation delay, on the basis of the characteristics of the vibration signal. Two bearing data sets, provided by Case Western Reserve University (CWRU)’s Bearing Data Center and the Center for Intelligent Maintenance Systems (IMS), University of Cincinnati, respectively, were used to verify the performance of the proposed algorithm. Compared with the LSTM-based methods and some CNN-based models, in our experiments the proposed algorithm took up less storage space and had shorter calculation delay under the same accuracy and noise immunity, and was more suitable for real-time fault diagnosis.

The rest of this paper is arranged as follows. In Section 2, the RNN variants and their applications are reviewed. Then, the LLRNN and its analysis are presented in Section 3. In the following Section 4, the experimental results using two data sets are illustrated. Finally, the conclusion is provided in Section 5.

## 2. Related Work

### 2.1. Application of RNN in Fault Detection

**Figure 2:**
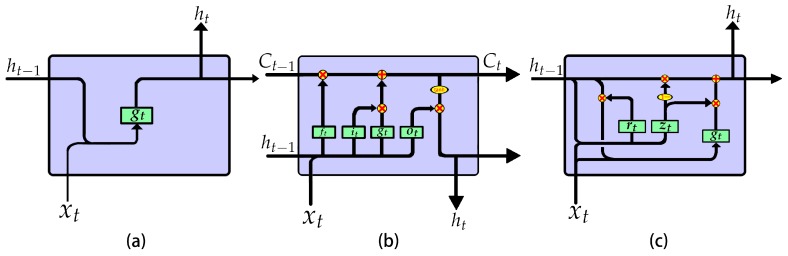
The inner structures of different recurrent neural network (RNN) cells: (**a**) RNN; (**b**) Long short-term memory network (LSTM); (**c**) Gated recurrent unit (GRU)

RNNs are mainly used to process sequence data, because they can store the feature information of historical data in the internal state (i.e., memory). Then, they combine current input data with memory and extract new features, as shown in Figure 2a. RNNs can be trained via backpropagation through time, but the vanishing gradient problem during backpropagation makes it difficult to capture long-term dependencies. The LSTM is an efficient RNN variant structure to solve this problem. It avoids long-term dependence problems through the gate mechanism and has the ability to extract long-term dependent feature information effectively [20]. The LSTM cell structure is shown in Figure 2b, and the calculation process is shown in Equation (1).

(1) $$\begin{array}{cc} f_{t} & =\sigma (W_{f}^{h}\cdot h_{t-1}+W_{f}^{x}\cdot x_{t}+b_{f})\\ i_{t} & =\sigma (W_{i}^{h}\cdot h_{t-1}+W_{i}^{x}\cdot x_{t}+b_{i})\\ g_{t} & =\tanh(W_{g}^{h}\cdot h_{t-1}+W_{g}^{x}\cdot x_{t}+b_{g})\\ o_{t} & =\sigma (W_{o}^{h}\cdot h_{t-1}+W_{o}^{x}\cdot x_{t}+b_{o})\\ C_{t} & =f_{t}⊙C_{t-1}+i_{t}⊙g_{t}\\ h_{t} & =o_{t}⊙\tanh\left(C_{t}\right) \end{array}$$

where $f_{t}$ denotes the forget gate, $i_{t}$ the input gate, $o_{t}$ the output gate, and $g_{t}$ the new candidate memory state. $h_{t-1}$ and $h_{t}$ denote the hidden states of the previous moment and the current moment. $x_{t}$ denotes the input of the current moment. $C_{t-1}$ and $C_{t}$ denote the memory states of the previous moment and the current moment. $W_{*}^{h}$ and $W_{*}^{x}$ denote the parameters related to $h_{t-1}$ and $x_{t}$ in the corresponding gate. and $b_{*}$ denotes the bias in the corresponding gate. “·” and “⊙” denote the operations of matrix multiplication and point multiplication, respectively.

The gate mechanism of LSTM is redundant, causing excessive parameters and calculations. In order to solve this problem, researchers have proposed several variants [21,22,23,24]. Gated recurrent unit (GRU) [24], the most successful variant, combines the forget gate and the input gate into an update gate and mixes the memory state and the hidden state. The GRU cell structure is shown in Figure 2c and the calculation process is shown in Equation (2):

(2) $$\begin{array}{cc} z_{t} & =\sigma (W_{z}^{h}\cdot h_{t-1}+W_{z}^{x}\cdot x_{t}+b_{z})\\ r_{t} & =\sigma (W_{r}^{h}\cdot h_{t-1}+W_{r}^{x}\cdot x_{t}+b_{r})\\ g_{t} & =\tanh(W_{g}^{h}\cdot \left(r_{t}⊙h_{t-1}\right)+W_{g}^{x}\cdot x_{t}+b_{g})\\ h_{t} & =\left(1-z_{t}\right)⊙h_{t-1}+z_{t}⊙g_{t}, \end{array}$$

where $r_{t}$ denotes the reset gate, controlling the influence level of $h_{t-1}$ on $g_{t}$. $z_{t}$ denotes the update gate, controlling the update of the memory state. The GRU can achieve performance comparable to LSTM with one less gate.

LSTMs have been successfully applied in many fields, and have also received much attention in the field of mechanical fault detection. Zhao et al. [25] combined a CNN with a bi-directional LSTM to propose a novel machine health monitoring system used for tool wearing prediction. Park et al. [26] employed an LSTM model in an edge device for industrial robot manipulator fault detection. Yuan et al. [27] applied an LSTM for aero engine fault diagnosis and remaining useful life (RUL) prediction. Zhang et al. [28] combined LSTM with Monte Carlo simulation for lithium-ion batteries’ RUL prediction. Cui et al. [29] combined fast Fourier transform (FFT) and RNN for bearing fault diagnosis. Wang et al. [30] applied LSTM to gear fault diagnosis. Liu et al. [31] proposed a GRU-based method for rolling bearings fault diagnosis by comparing the reconstruction errors generated from multiple-dimension time-sequence data. This paper proposes a lightweight model with low delay based on a special LSTM cell structure for rotating mechanism fault diagnosis.

### 2.2. Introduction of JANET

**Figure 3:**
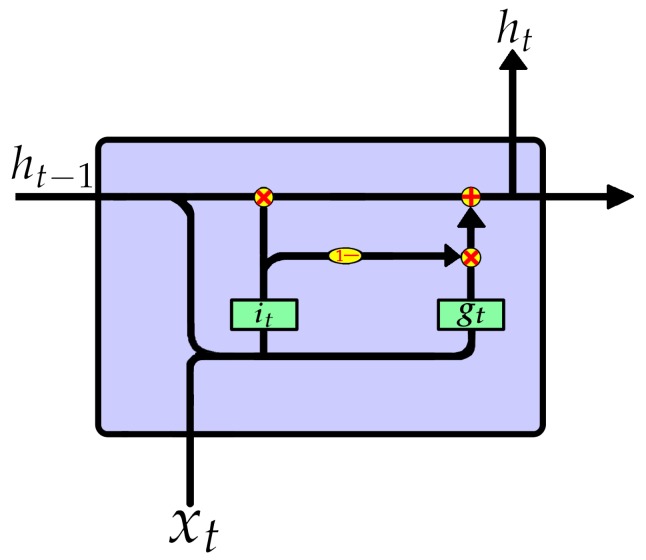
The inner structures of JANET.

Der Westhuizen et al. [32] proposed a new LSTM cell structure with only one forget gate, namely, “just another network” (JANET). Not only does JANET provide computational savings, but also outperforms the standard LSTM on multiple benchmark data sets and competes with some of the best contemporary models. The JANET cell structure is shown in Figure 3, and the calculation process is shown in Equation (3).

(3) $$\begin{array}{cc} f_{t} & =\sigma (W_{f}^{h}\cdot h_{t-1}+W_{f}^{x}\cdot x_{t}+b_{f})\\ g_{t} & =\tanh(W_{g}^{h}\cdot h_{t-1}+W_{g}^{x}\cdot x_{t}+b_{g})\\ C_{t} & =f_{t}⊙C_{t-1}+\left(1-f_{t}\right)⊙g_{t}\\ h_{t} & =C_{t} \end{array}$$

JANET retains the most important forget gate $f_{t}$ in LSTM. Since $f_{t}$ determines which information should be discarded, the information that $f_{t}$ does not suggest to drop should be retained. Therefore, $\left(1-f_{t}\right)$ is approximately regarded as $i_{t}$, reducing both the parameters and calculation caused by the input gate. The output gate selects the useful information in the memory state $C_{t}$ and passes it to the hidden state $h_{t}$. In fact, this task can be handed over to the forget gate of the next moment. Based on this idea, JANET cancels the output gate and merges the hidden state $h_{t}$ and memory state $C_{t}$, just like the GRU. JANET has a simpler structure and less calculation.

## 3. The Proposed Method

### 3.1. Model Structure

**Figure 4:**
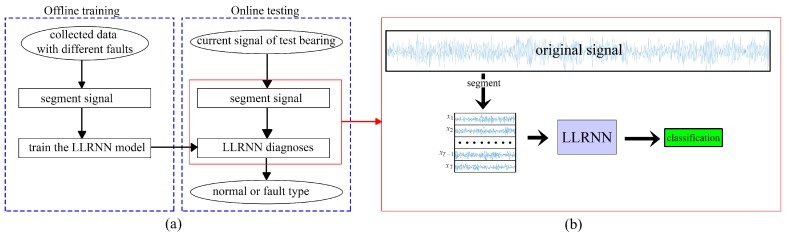
(**a**) Flowchart of the proposed method. (**b**) Diagnostic process of the proposed method. LLRNN: low-delay lightweight recurrent neural network.

In this paper, a low-delay lightweight recurrent neural network (LLRNN) model for rotating machinery fault diagnosis is designed based on a JANET cell, and the overall flowchart is shown in Figure 4a. The input vibration signal is segmented into several shorter sub-signals. Then, these sub-signals are sent to the network directly and converted into the final classification results, as shown in Figure 4b. As an end-to-end model, the proposed model can turn the data collected by sensors into the finally desired prediction results without any manual participation, which could be used for real-time monitoring and has two improvements: (1) It is based on a simpler cell structure with fewer parameters, and the proposed model consumes less storage space while maintaining its performance. (2) Segmenting the signal before sending it into the network not only reduces the training difficulty of the network, but also improves the noise immunity.

### 3.2. Network Structure

**Figure 5:**
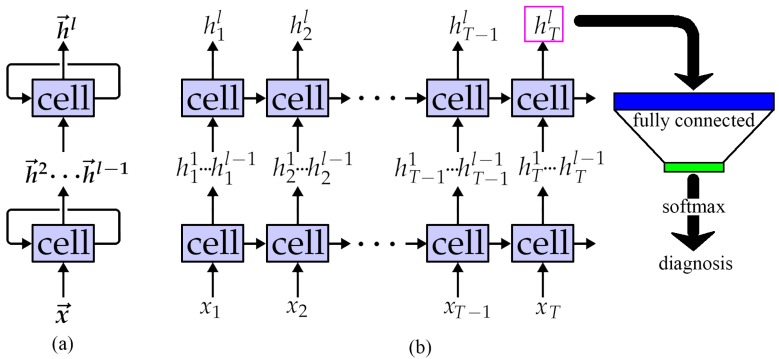
LLRNN network structure: (**a**) The real structure; (**b**) The logically expanding structure.

Using the output of the previous layer as the input of next layer, an RNN can also be stacked to form a deeper structure similar to a CNN, as shown in Figure 5a. The feature extraction ability of the model can be improved and the learned features have more semantic information. However, this operation brings a risk of over-fitting and more calculation. After logically expanding, the actual calculation process of an *l*-layer RNN is described in Figure 5b. The length of each $x_{t}$ is $D_{x}$, and *T* = steps = *L*/$D_{x}$. In subsequent experiments, the effects of various parameters on the performance of the model are verified, including accuracy, noise immunity and computational delay.

### 3.3. Model Analysis

#### 3.3.1. The Parameters and Calculation of Different Cell Structures

As shown in Equation (3), the calculation process of each gate is essentially a fully connected layer. Let $D_{h}$ denote the quantity of hidden units in each gate, and $D_{x}$ denote the input data dimension. The parameters and calculation cost are defined as in Equations (4) and (5):

(4) $$\begin{array}{cc} Params_{gate} & =\left(D_{h}+D_{x}\right)\cdot D_{h}+D_{h} \end{array}$$

(5) $$\begin{array}{cc} FLOPs_{gate} & =2\cdot \left(D_{h}+D_{x}\right)\cdot D_{h} \end{array}$$

where $Params_{gate}$ and $FLOPs_{gate}$ (FLOP: floating-point operations) denote the parameters and calculation amount of each gate, respectively.

The parameter quantity of the cell is the sum of that of all the gates inside it. LSTM has four gates, GRU has three, and JANET has only two. Their parameters are shown in Equations (6)–(8). JANET has only half the number of parameters that LSTM does, and two-thirds those of GRU:

(6) $$\begin{array}{cc} Params_{LSTM} & =4\cdot Params_{gate} \end{array}$$

(7) $$\begin{array}{cc} Params_{GRU} & =3\cdot Params_{gate} \end{array}$$

(8) $$\begin{array}{cc} Params_{JANET} & =2\cdot Params_{gate} \end{array}$$

The calculation of the cell in each moment equals to the sum of the that of all the gates, plus the calculation cost of information interaction between the respective gates, which essentially are some point addition and point multiplication operations. LSTM has four multiplication interactions, GRU has five, and JANET has four. The total calculations are shown in Equations (9)–(11):

(9) $$\begin{array}{cc} FLOPs_{LSTM} & =(4\cdot FLOPs_{gate}+4\cdot D_{h})\cdot steps \end{array}$$

(10) $$\begin{array}{cc} FLOPs_{GRU} & =(3\cdot FLOPs_{gate}+5\cdot D_{h})\cdot steps \end{array}$$

(11) $$\begin{array}{cc} FLOPs_{JANET} & =(3\cdot FLOPs_{gate}+4\cdot D_{h})\cdot steps \end{array}$$

where steps denotes the number of sub-segments of a sequence signal, and steps = *L*/$D_{x}$, as shown in Figure 1.

#### 3.3.2. Analysis of Step Length

As shown in Equations (8) and (11), $D_{h}$ and $D_{x}$ affect both the calculation and parameters, and steps affects only the calculation. When the signal length *L* of the input model is fixed, steps = *L*/$D_{x}$ and the Equations (12) and (13) can be obtained by substituting it into Equations (8) and (11):

(12) $$\begin{array}{cc} Params_{JANET} & =2\cdot D_{h}\cdot D_{x}+2\cdot D_{h}\cdot \left(D_{h}+1\right) \end{array}$$

(13) $$\begin{array}{cc} FLOPs_{JANET} & =\frac{4\cdot D_{h}\cdot \left(D_{h}+1\right)}{D_{x}}\cdot L+4\cdot D_{h}\cdot L \end{array}$$

Equation (12) shows that the total parameters of the model are positively correlated with $D_{x}$, and the occupancy of parameters and storage space increases with $D_{x}$. On the contrary, Equation (13) shows that the total calculation of the model is negatively correlated with $D_{x}$, and the increment of $D_{x}$ will reduce the calculation. In addition to the computation, the waiting time caused by data dependencies also affects the total computing time of the model. The RNN has a serious problem of calculation delay when processing long sequence data. Because the calculation of $h_{t}$ must wait for $h_{t-1}$, the parallel computing power of the GPU cannot solve it. When training the network using a vibration signal $\vec{x}=\left\{x_{1},x_{2},\ldots ,x_{t},\ldots ,x_{L}\right\}$, the model takes a long time to process such a long time sequence if every point is used as the input of each moment. It is difficult to train the network. In addition, there is too little effective information that can be extracted, and the extracted features are seriously disturbed by noise if the input vibration signal is single-point. Noise interference not only changes the amplitude information of the signal, but may even change the direction information of the vibration. As a result, the accuracy of the algorithm is reduced.

In order to solve these problems, we segment the vibration signal of length L into steps-segment sub-signals of length $D_{x}$, as shown in Figure 1. When $D_{x}$-point vibration information is used as the input of each moment, the step number of the time sequence will reduce from *L* to *L*/$D_{x}$ and the total calculation decreases according to Equation (13).

Both the fault shock signal and noise signal are superimposed on the normal signal. The operation of each gate in the cell is a vector multiplied by a matrix, which is essentially a weighted summation of each point of input. It plays a certain smoothing effect, the same as a mean operation, as shown in Equation (14):

(14) $$\begin{array}{ccccc} S_{data} & = & S_{vibration}+S_{noise}\\ S_{vibration} & = & S_{nornaml}+S_{fault}\\ Operation_{gate} & = & \sum_{i}^{D_{x}}w\left(i\right)\cdot S_{nornaml}\left(i\right) & +\sum_{i}^{D_{x}}w\left(i\right)\cdot S_{fault}\left(i\right) & +\sum_{i}^{D_{x}}w\left(i\right)\cdot S_{noise}\left(i\right) \end{array}$$

where $S_{data}$ denotes the signal of input data, $S_{vibration}$ and $S_{noise}$ denote the signal of vibration part and noise part in $S_{data}$, and $S_{normal}$ and $S_{fault}$ denote the signal of the normal part and the fault part in $S_{vibration}$. Most noise is subject to the zero-mean distribution and $E[S_{noise}]=0$. This means that the larger $D_{x}$ is, the closer $\sum_{i}^{D_{x}}w\left(i\right)\cdot S_{noise}\left(i\right)$ is to 0. Although the $E[S_{fault}]\neq 0$, for the most part the value of $S_{fault}$ is 0, except for the moments where a fault shock happens. If $D_{x}$ is too large, $\sum_{i}^{D_{x}}w\left(i\right)\cdot S_{noise}\left(i\right)$ will be very small. It is difficult for a model to extract the feature of faults. Consequently, the $D_{x}$ should be appropriate—not too large or too small. These analyses were verified in the subsequent experiments.

## 4. Experiment and Analysis

Two bearing data sets were used to verify the performance of the proposed algorithm and the previous analyses. The data sets were provided by Case Western Reserve University (CWRU)’s Bearing Data Center [33] and the Center for Intelligent Maintenance Systems (IMS), University of Cincinnati [34]. The hardware resources of the computing device used for the experiment were as follows, CPU: Intel Core i7-8700k, 3.7 GHz, six-core twelve threads; GPU: NVIDIA 1080Ti, 11G ×2; Memory: 32 GB; Storage: 2 TB.

The software environment was as follows, Ubuntu 16.04 system; TensorFlow 1.10 framework, Python programming language.

### 4.1. The Impact of Model Structure on Performance

#### 4.1.1. Introduction of the CWRU Data Set

**Figure 6:**
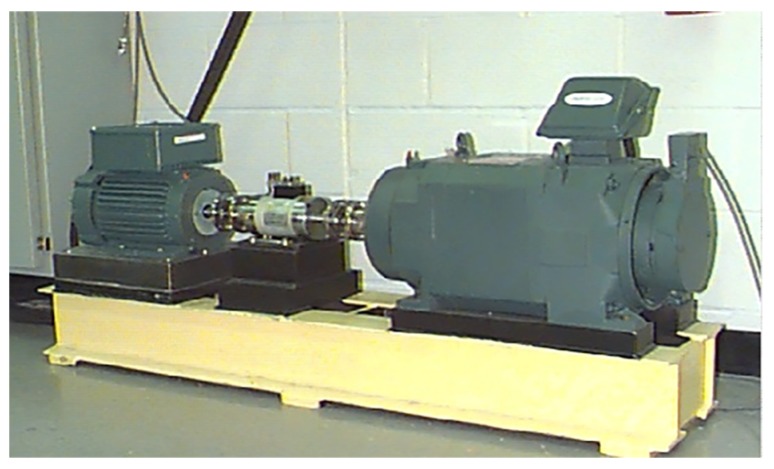
Fault simulation test rig of the Case Western Reserve University (CWRU) data set.

To validate the previous analyses, 12-kHz drive-end data collected by Case Western Reserve University (CWRU)’s Bearing Data Center were used. Figure 6 shows the test rig used for data collection. The data set contains four different categories, namely, normal bearings, bearings with a faulty ball (ball), bearings with a faulty inner race (inner) and bearings with a faulty outer race (outer). For each type of fault, there are three fault diameters, 0.007 inch, 0.014 inch and 0.021 inch, respectively. Thus, there are 10 classifications in this dataset.

**Figure 7:**
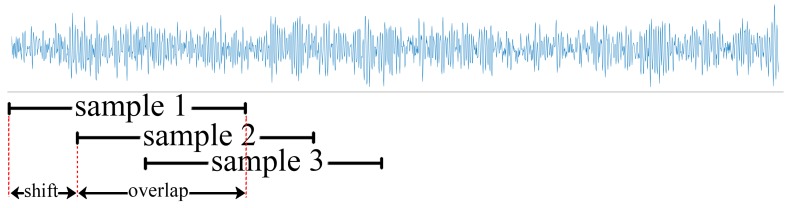
Diagram of overlapping sampling.

Due to the limited experimental data, the overlapping sampling method was used to enhance the data according to references [35,36], as shown in Figure 7.

The data set was divided into four subsets, corresponding to four different loads, namely load 0, load 1, load 2, and load 3. As shown in Table 1, every category of data subset under each load contained 800 training samples, 100 test samples, and 100 validation samples, for a total of 8000 training samples, 1000 test samples, and 1000 validation samples.

Under normal circumstances, bearing vibration signals are affected by the surrounding ambient noise. The CWRU dataset selected in this study was collected in an environment with a relatively low level of ambient noise and therefore cannot reflect the performance of the fault diagnosis algorithm in an actual environment. In addition, there are a number of noise sources in an actual environment, and it is impossible to obtain training samples under all the conditions in various noise environments. Therefore, noise was added to the samples in the raw test set to simulate data from actual conditions. Using the resultant data for testing could produce results closer to those obtained under actual industrial production conditions. Accordingly, white Gaussian noise from 10 dB to −4 dB was added to the data to simulate actual conditions. Signal-to-noise ratio (SNR) is defined as in Equation (15), and was used when adding noise:

(15) $$SNR=10\log_{10}\left(\frac{P_{signal}}{P_{noise}}\right)$$

where ***P***${}_{signal}$ and ***P***${}_{noise}$ are the intensities of the raw and noise signals, respectively.

To examine the noise immunity of the proposed algorithm, the learning model with the highest accuracy for the validation set in 1000 iterations was selected. Noise-containing data were randomly generated 10 times for each sample in the test set of the dataset, and were used for testing and statistically analysing the experimental results, which were represented in the form of the mean.

#### 4.1.2. Experimental Results and Analysis

To verify the analysis of the impact of step length on performance in Section 3.3.2, the data subset under load 0 was selected for experimentation, with the network structure having 128 hidden units and 1 layer. We repeated the experiment 10 times with each parameter configuration, and calculated the mean value of the results, as shown in Table 2.

As the $D_{x}$ increased, the validation accuracy and test accuracy slightly increased. When $D_{x}$ exceeded 256, the accuracy began to decrease due to the smoothing effect caused by the lengthening of $D_{x}$, which made the model less sensitive to the detail change information of the vibration signal. When $D_{x}$ increased from 512 to 1024, the increment of noise immunity was greater than the decrement of feature extraction capability, so the noise immunity ability rebounded. The $D_{x}$ was set to 64 to preserve the noise immunity of the model.

The number of hidden units determines the ability of the RNN model to extract feature information; too few leads to under-fitting, and too many causes over-fitting. The extracted feature is finally transformed into a vector, which represents the category, through a fully connected layer as shown in Figure 4b. This process also has a smoothing effect and an impact on noise immunity. As shown in Table 3, as the number of hidden units increased, the test accuracy first rose and then fell. The reason for this is that the generalisation ability increased with the complexity of the model, then decreased due to over-fitting when the number of cells exceeded 128. Unlike the impact of $D_{x}$, the decrement of noise immunity was not due to the reduced feature extraction capabilities, but to the over-fitting to noise-free training data.

When hidden units increased from 16 to 256, the theoretical calculation increased by 50 times, but the real training time only increased by less than three percent, and the test time nearly doubled. These gaps were caused by the following two reasons: (1) The increment of hidden units only brought the calculation for each moment without increasing the waiting time. (2) The GPU has parallel computing power; 16 units or 256 units were calculated synchronously, and the time consumed was the same as that for calculating only one unit. Only when calculating the sum of all the results of each unit did the calculating time increase slightly.

RNNs can also be stacked like CNNs to build deeper models, as shown in Figure 5b. The number of hidden units in the network was set to 128 for the experiment due to the highest noise immunity performance, and the effects of different layer numbers on the model performance are shown in Table 4.

The model had the highest test accuracy and noise immunity at two layers due to the appropriate model complexity. The model with more layers had lower performance caused by over-fitting, and both space occupancy and calculation delay increased without any increment of test accuracy or noise immunity.

As shown in the above experiments, all the analyses of the impact of network structure and step length on performance were verified. As $D_{x}$ increased, the calculation delay decreased, the accuracy first rose then fell, and the noise immunity increased. As $D_{h}$ increased, the calculation delay on the CPU decreased, and both the accuracy and noise immunity increased then decreased. As the number of layers increased, the calculation delay increased, and both the accuracy and noise immunity increased then decreased. The trend of performance was almost the same as assumed in Section 3. The proposed method had the most satisfactory result for fault diagnosis on the CWRU data set in the condition that $D_{x}$ was equal to 64, $D_{h}$ was equal to 128, and layer was equal to 2.

### 4.2. The Universality of the Proposed Method

In the previous experiments, the analyses of the impact of network parameters on the performance were verified, and the proposed method had a satisfactory result on the CWRU data set. However, the mechanical system in the CWRU bearing data experiment was relatively simple, and the fault was also caused by human damage, whose vibration characteristics may be different from that of natural wear in an actual production environment. To verify the universality of the model, extra experiments were performed using the IMS data set, which was collected in a more complex mechanical system with natural wear faults. LSTM and GRU were used for experimental comparison to verify whether the simplification of the cell structure would reduce the network performance.

#### 4.2.1. Introduction of the IMS Data Set

**Figure 8:**
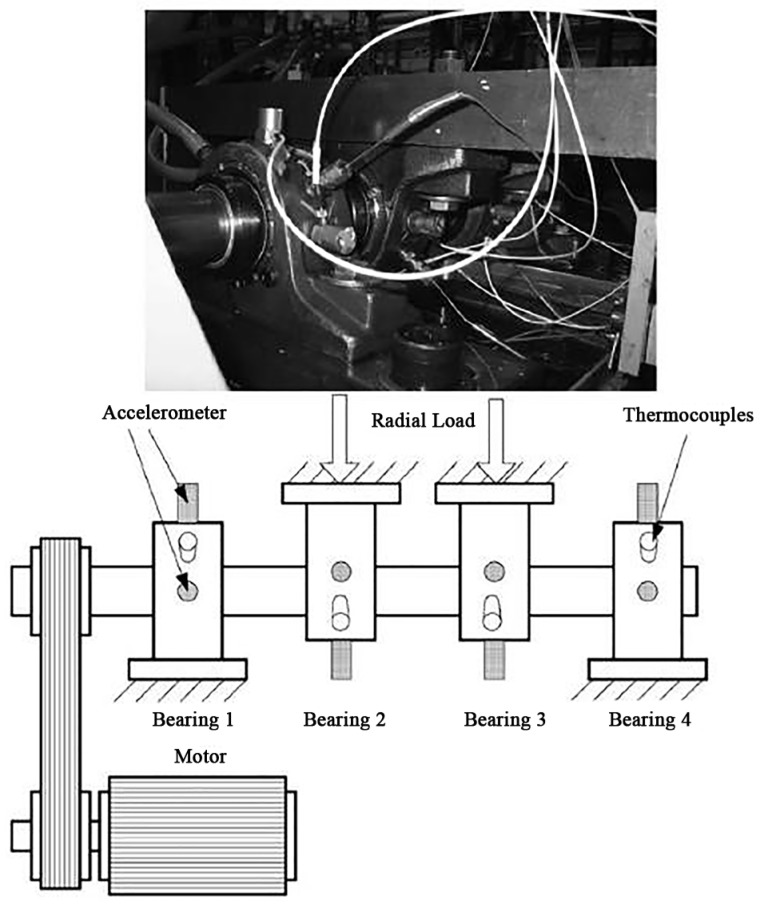
Test rig and sensor placement of the Center for Intelligent Maintenance Systems (IMS) dataset.

This dataset was provided by the Center for Intelligent Maintenance Systems (IMS), University of Cincinnati, and shared on the website of the Prognostic Data Repository of NASA [37]. The structure of the mechanical system is shown in Figure 8, and the data records the entire wear process of the bearing.

**Figure 9:**
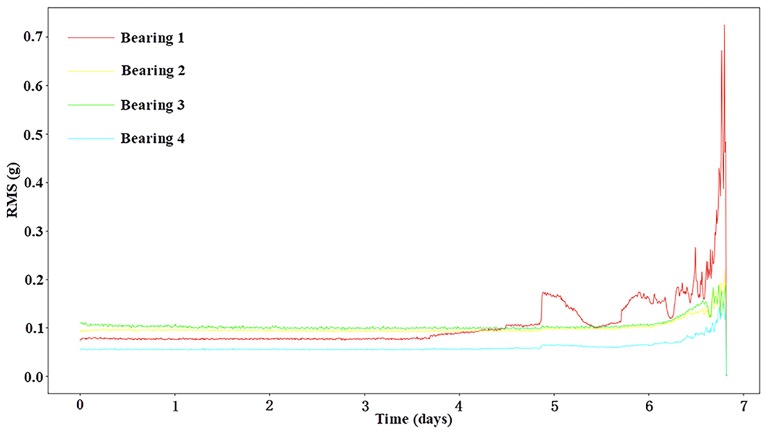
The root mean square (RMS) values in dataset 2 of IMS.

The bearings experienced “increase–decrease–increase” degradation trends. This behaviour was due to the “self-healing” nature of the damage [38]. First, the amplitude of vibration increased because of the impact caused by the initial surface defect (e.g., spalling or cracks). Then, the initial defect was smoothed by continuous rolling contact, leading to the decrease of the impact amplitude. Finally, the damage spread over a broader area, and the vibration amplitude increased again. During the self-healing period, the amplitude of the fault bearing was similar to that of the normal bearing, making it difficult to detect the fault during the self-healing period, as shown in Figure 9.

At the end of the experiment, the inner race defect, outer race defect, and roller element defect were detected manually [34]. As shown in Figure 9, the red curve indicates the wear process of the outer race defect in bearing 1. The self-healing appeared after the failure on the fifth day, and its amplitude was basically the same as that of the normal bearing (green curve). In order to increase the difficulty of diagnosis, we chose the bearings in the self-healing period as fault data, and the normal bearing with similar amplitude as normal data. Also, a length of 1024 was directly sampled as a sample instead of overlapping sampling due to the relatively sufficient size of the IMS data set. The fault data categories are shown in Table 5.

#### 4.2.2. Experimental Results and Analysis

In order to verify whether the simplification of the cell structure had too much of a negative impact on performance, a comparison experiment was performed using GRU and LSTM with the same number of hidden units and almost-calculated quantities.

The comparison results of the CWRU data set are shown in Table 6 and Table 7. When the number of hidden units was 128 and the number of layers was 1, neither the test accuracy nor noise immunity of GRU${}_{1}$ and LSTM${}_{1}$ were improved much compared with LLRNN. However, the parameter quantity of GRU${}_{1}$ increased by one-half and that of LSTM${}_{1}$ increased by one time, the test delay of GRU${}_{1}$ increased by 50%, and that of LSTM${}_{1}$ increased by 25%. The GRU${}_{1}$ had less calculation than the LSTM${}_{1}$ but the calculation delay of GRU${}_{1}$ was higher than that of LSTM${}_{1}$. This is because the four gates of the LSTM${}_{1}$ were independent and could be calculated in parallel, while the calculation of $g_{t}$ in GRU${}_{1}$ must wait for the output of $r_{t}$, as shown in Figure 2c, resulting in more calculation time than LSTM${}_{1}$.

Under a structure of one layer with parameter quantity and calculation amount similar to LLRNN, the performances of GRU${}_{2}$ and LSTM${}_{2}$ were slightly lower than that of LLRNN, while the delay of LSTM${}_{2}$ was 13% higher and that of GRU${}_{2}$ was 30% higher. In the cell structure work flow, the calculation of the LSTM${}_{2}$ activation function tanh brought calculation and computational delays, resulting in the calculation time of LSTM${}_{2}$ being longer than that of LLRNN. Under the structure of two layers with the parameter quantity and calculation amount similar to LLRNN, the performances of GRU${}_{3}$ and LSTM${}_{3}$ were improved compared with GRU${}_{2}$ and LSTM${}_{2}$. Although the performance was similar to that of LLRNN, the delay of LSTM${}_{3}$ increased by 24% and that of GRU${}_{3}$ increased by 37%. As shown in Table 6, CNN-based models [12,13,14,15] gained competitive performance in no-noise test data, but they took up more storage and consumed more computing time due mainly to the over-complexity of the network structure. Although reference [14] had similar parameters to LLRNN, its theoretical calculation was six times greater and its real computing time was approximately ten times greater compared with the proposed method. Besides, the accuracies of CNN models fell rapidly in the noisy environment while the accuracy of LLRNN still exceeded 90%, as shown in Table 7. The model structure and training method of SVM both differ from those for neural networks, so it is meaningless to discuss the training time of reference [6]. Not only were the test accuracies and noise immunities of SVM much lower than those of LLRNN, but also the test time of the former was dozens of times greater than that of the latter. The primary reason is that the multi-category task of SVM is broken down into several binary tasks, which results in extra computation.

The experimental results of the IMS data set are shown in Table 8. The self-repair of the mechanical system made the data characteristics of the faulty bearing similar to those of the normal bearing. Although the test accuracy gained satisfactory results, the complexity of the system made the data more difficult to distinguish in high-noise environments. The test accuracy in the simulation environment of 0 dB was lower than that of the CWRU data set at least 6%. Like the experimental results of the CWRU dataset, the performance of LLRNN was not worse than any structure using LSTM or GRU.

In contrast to the experimental results of the CWRU data, two CNN models gained competitive noise immunity. However, their computing time was still much higher than that of proposed method. The other two still could not work in a noisy environment—especially reference [14], whose prediction accuracy was similar to random guess in a severely noisy environment, at around 25%. The SVM model also behaved poorly; its performance was similar to that in the CWRU data set. However, its test time was much lower than that in the CWRU data set. Because there are four categories of IMS data and the quantity of decomposed binary tasks is only four-tenths that of CWRU, it had much less calculation. According to the results of the above experiments, the ingenious simplification of the JANET cell did not cause serious performance degradation. The calculation delay of LLRNN was at least 10% lower than any network structure using LSTM or GRU while maintaining a satisfactory performance. Compared with some CNN and SVM models, the proposed method not only gained higher prediction accuracy and noise immunity, but also spent the least amount of computing time.

## 5. Conclusions

In this paper, a low-delay lightweight recurrent neural network (LLRNN) model is proposed for mechanical fault diagnosis, which is an end-to-end processing model. From input data to output diagnostics, the process executes automatically without any manual involvement. Thus, the diagnostic quality is not dependent on expert experience. According to the work flow of the JANET cell structure, this paper analysed the influence of several factors (e.g., $D_{x}$, steps, $D_{h}$, and layers) on the performance of the model, including accuracy, noise immunity, and calculation delay. The relationship between these factors and performance was verified in the experiment. The proposed method obtained the highest accuracy and noise immunity performance when layer = 2, $D_{x}$ = 64, and $D_{h}$ = 128. This could give some guidance to model design in related fields. The experimental results of the two data sets CWRU and IMS prove that the simplified structure of LLRNN could achieve performance comparable to any network model using LSTM or GRU, and the computational delay decreased by at least 10%, which is more suitable for real-time fault detection systems.

The proposed method still requires some improvements due to the data dependencies of the RNN work flow. As shown in Figure 5b and Equation (3), the calculation of $h_{t}$ must wait for $h_{t-1}$. Let $t_{gate}$ denote the time consumed in calculating each gate. Although the GPU has parallel computing power, it could only calculate each gate synchronously with the limitation of data dependencies. Ignoring the consumption of communication between each gate, the time consumed each moment could approximate $t_{gate}$. When dealing with time series data with length of steps, the GPU must wait steps times caused by the data dependencies, so the time of calculating each data sample is $t_{gate}\times$steps. If the dependence between $h_{t}$ and $h_{t-1}$ could be eliminated, the calculation of each moment could be performed independently and the GPU could fully utilise its parallel computing power without any waiting. The calculation of steps moments could be dealt with at the same time. Consequently, the time of calculating each data sample could be further reduced to approximately $t_{gate}$. Now that the CPU of the edge device is basically multi-core and has certain parallel computing capabilities, it means that the test time could decrease as well.

## Figures and Tables

**Table 1:** Classification of the CWRU bearing fault data subsets under each load.

Training Samples	Validation Samples	Test Samples	Fault Types (Inches)	Labels
800	100	100	Normal	1
800	100	100	Ball 0.007	2
800	100	100	Ball 0.014	3
800	100	100	Ball 0.021	4
800	100	100	Inner 0.007	5
800	100	100	Inner 0.014	6
800	100	100	Inner 0.021	7
800	100	100	Outer 0.007	8
800	100	100	Outer 0.014	9
800	100	100	Outer 0.021	10

**Table 2:** Experimental results for the effects of different step lengths.

$D_{x}$	Params (KB)	FLOPs ($10^{5}$)	Training Time	Test Time (s)	Validation Acc(%)	Test Acc (%)	10 dB Acc (%)	5 dB Acc (%)	0 dB Acc (%)	−4 dB Acc (%)
1	130	682	6 h 33 m 29 s	2.562	97.1	95.9	84.4	60.5	44.0	18.4
2	131	343	3 h 01 m 48 s	1.194	97.8	96.6	94.1	80.7	47.3	21.6
4	133	174	1 h 32 m 12 s	0.552	98.6	97.1	94.4	81.6	52.4	28.1
8	137	89.8	46 m 14 s	0.312	99.2	98.2	94.8	82.9	56.2	29.2
16	145	47.5	23 m 59 s	0.184	99.3	99.1	98.6	97.3	85.5	49.6
32	161	26.4	13 m 04 s	0.097	99.3	99.3	98.3	97.5	91.0	68.6
64	193	15.8	7 m 58 s	0.054	99.4	99.3	99.0	97.8	91.8	75.5
128	257	10.5	4 m 39 s	0.050	99.4	99.4	98.9	97.2	89.9	74.1
256	385	7.88	3 m 48 s	0.048	99.4	98.9	98.3	96.3	88.8	73.5
512	641	6.56	2 m 30 s	0.048	96.9	96.5	95.6	93.4	85.9	71.2
1024	1153	5.90	2 m 13 s	0.048	96.2	96.0	95.7	94.9	90.7	79.3

Training time denotes the time of training the network on the GPU with batch size equaling 100; Test time denotes the time of testing the network on the CPU using all the test samples of load 0.

**Table 3:** Experimental results for the effects of different hidden units.

$D_{h}$	Params (KB)	FLOPs ($10^{4}$)	Training Time (s)	Test Time (s)	Validation Acc (%)	Test Acc (%)	10 dB Acc (%)	5 dB Acc (%)	0 dB Acc (%)	−4 dB Acc (%)
16	5.06	4.10	471	0.047	99.2	98.1	95.4	87.9	70.6	47.4
32	12.13	9.83	471	0.048	99.4	98.9	97.9	94.0	82.4	58.9
48	21.19	17.2	472	0.051	99.4	99.1	98.7	96.7	87.7	67.0
64	32.25	26.2	473	0.051	99.3	99.2	98.6	96.6	88.7	69.5
80	45.31	36.9	475	0.053	99.4	99.2	98.8	97.1	89.5	70.9
96	60.38	49.2	476	0.053	99.4	99.2	98.8	97.2	90.1	72.7
128	96.50	78.6	478	0.054	99.3	99.3	99.0	97.8	91.8	75.5
160	140.63	115	480	0.063	99.4	99.2	98.8	97.4	90.4	74.0
192	192.75	157	482	0.071	99.3	99.1	98.8	97.5	90.3	73.9
224	252.88	206	485	0.075	99.3	99.0	98.8	97.5	90.6	73.8
256	321.00	262	486	0.090	99.1	99.0	98.7	97.6	90.6	73.5

**Table 4:** Experimental results for the effects of different numbers of layers.

Layer	Params (KB)	FLOPs ($10^{6}$)	Training Time (s)	Test Time (s)	Validation Acc (%)	Test Acc (%)	10 dB Acc (%)	5 dB Acc (%)	0 dB Acc (%)	−4 dB Acc (%)
1	193	1.58	478	0.054	99.3	99.3	99.0	97.8	91.8	75.5
2	386	3.16	684	0.076	99.5	99.3	99.1	98.4	93.6	79.0
3	579	4.74	1126	0.103	99.3	99.0	98.6	97.3	90.6	72.8
4	772	6.32	1332	0.123	99.0	98.7	98.1	96.6	89.8	73.2
5	965	7.91	1792	0.146	98.3	98.0	97.4	96.1	89.6	73.4

**Table 5:** Classification of the IMS bearing fault data set.

Training	Validation	Test	Fault Types	Labels
1600	200	200	Normal	1
1600	200	200	Roller	2
1600	200	200	Outer	3
1600	200	200	Inner	4

**Table 6:** Experimental results of various methods in the CWRU dataset.

Method	Layer	$D_{h}$	Params (KB)	FLOPs ($10^{6}$)	Training Time (s)	Test Time (s)	Load 0 Acc (%)	Load 1 Acc (%)	Load 3 Acc (%)	Load 4 Acc (%)
LLRNN	1	128	193	1.58	478	0.054	99.3	99.7	99.8	99.7
GRU${}_{1}$	1	128	289	2.37	518	0.081	99.2	99.2	99.5	99.6
LSTM${}_{1}$	1	128	386	3.15	486	0.068	99.7	99.7	99.8	99.8
GRU${}_{2}$	1	100	193	1.58	502	0.070	99.2	99.1	99.2	99.4
LSTM${}_{2}$	1	84	195	1.60	467	0.061	99.2	99.4	99.7	99.6
GRU${}_{3}$	2	64	193	1.58	987	0.074	99.3	99.3	99.6	99.6
LSTM${}_{3}$	2	53	195	1.59	788	0.067	99.4	99.5	99.7	99.7
Reference [12]	-	-	50371	145	876	0.865	99.6	99.6	99.7	99.8
Reference [13]	-	-	565	80.8	586	0.584	99.7	99.6	99.8	99.7
Reference [14]	-	-	206	10.1	873	0.515	99.8	99.7	99.7	99.8
Reference [15]	-	-	367	200	2992	5.752	99.7	99.6	99.8	99.7
Reference [6]	-	-	-	-	37	7.288	82.3	83.5	85.8	88.3

LLRNN denotes the network structure using JANET cell with $D_{x}$ = 64, $D_{h}$ = 128, and layer = 1. GRU${}_{1}$ and LSTM${}_{1}$ denotes the network structure using GRU and LSTM cells with the same $D_{x}$, $D_{h}$ and layer as LLRNN. GRU${}_{2}$ and LSTM${}_{2}$ denote the network structure using GRU and LSTM cells under layer = 1 and $D_{x}$ = 64 with approximately the same amount of calculation as LLRNN. GRU${}_{3}$ and LSTM${}_{3}$ denote the network structure using GRU and LSTM cells under layer = 2 and $D_{x}$ = 64 with approximately the same amount of calculation as LLRNN.

**Table 7:** Noise immunity of various methods in the CWRU dataset.

Method	load 0 Acc (%)			load 1 Acc (%)			load 2 Acc (%)			load 3 Acc (%)		
	10 dB	5 dB	0 dB	10 dB	5 dB	0 dB	10 dB	5 dB	0 dB	10 dB	5 dB	0 dB
LLRNN	99.0	97.8	91.5	99.5	98.7	94.0	99.7	99.4	95.1	99.5	98.3	91.2
GRU${}_{1}$	99.0	97.9	91.8	99.2	98.9	94.1	99.5	99.0	91.8	99.6	98.9	90.3
LSTM${}_{1}$	99.1	98.1	92.1	99.5	99.3	93.5	99.9	99.7	93.1	99.7	99.7	91.5
GRU${}_{2}$	98.1	97.1	89.9	99.0	98.5	91.4	99.1	98.1	91.5	99.1	97.7	89.1
LSTM${}_{2}$	98.3	96.7	89.5	99.3	98.5	91.6	99.2	98.7	92.1	99.0	98.2	89.3
GRU${}_{3}$	99.0	97.8	91.1	99.2	98.5	92.5	99.3	98.8	93.0	99.3	98.4	91.5
LSTM${}_{3}$	99.1	97.8	90.6	99.3	98.7	92.2	99.4	99.1	94.4	99.3	98.5	92.7
Reference [12]	92.9	78.3	33.3	93.5	82.4	35.6	95.9	86.3	37.5	96.9	88.3	38.3
Reference [13]	97.5	92.4	56.0	98.2	93.4	57.4	98.5	94.4	57.8	98.9	95.2	58.1
Reference [14]	96.2	86.4	61.2	96.7	87.6	61.7	97.1	88.4	62.1	97.2	88.7	63.8
Reference [15]	77.1	54.8	36.9	78.6	58.1	35.3	75.3	55.8	37.9	79.6	54.6	39.2
Reference [6]	80.5	77.4	43.8	81.5	73.4	40.5	80.5	77.4	43.8	81.5	74.8	42.3

**Table 8:** Experimental results of various methods in the IMS dataset.

Method	Layer	$D_{h}$	Params (KB)	FLOPs ($10^{6}$)	Training Time (s)	Test Time (s)	Test Acc (%)	10 dB Acc (%)	5 dB Acc (%)	0 dB Acc (%)	−4 dB Acc (%)
LLRNN	1	128	193	1.58	360	0.052	99.5	99.1	97.6	85.2	63.6
GRU${}_{1}$	1	128	289	2.37	462	0.075	99.6	99.7	98.6	84.2	60.8
LSTM${}_{1}$	1	128	386	3.15	373	0.063	99.6	99.3	97.9	85.1	63.7
GRU${}_{2}$	1	100	193	1.58	462	0.067	99.3	98.6	98.4	82.4	59.0
LSTM${}_{2}$	1	84	195	1.60	357	0.056	99.3	98.3	97.0	84.5	62.4
GRU${}_{3}$	2	64	193	1.58	790	0.062	99.5	98.8	98.0	83.8	59.2
LSTM${}_{3}$	2	53	195	1.59	638	0.058	99.6	99.5	98.3	85.5	63.6
Reference [12]	-	-	50,371	145	1006	1.344	99.7	98.5	97.0	87.1	60.3
Reference [13]	-	-	565	80.8	615	0.473	99.8	97.9	95.4	87.2	61.2
Reference [14]	-	-	206	10.1	723	0.436	99.8	89.6	60.6	26.1	25.3
Reference [15]	-	-	367	200	2441	4.657	99.7	96.6	82.4	61.1	35.6
Reference [6]	-	-	-	-	32	3.497	76.0	72.8	57.7	50.2	28.9

## Abbreviations

LLRNN	Low-Delay Lightweight Recurrent Neural Network
LSTM	Long Short-Term Memory Network
AutoEncoder	Automatic Encoder
DT-CWT	Dual Tree-Complex Wavelet Transform
CWTS	Continuous Wavelet Transform Scalogram
AI	Artificial Intelligence
CHMM	Coupled Hidden Markov Model
k-NN	k-Nearest Neighbour
WPT	Wavelet Packet Transform
SVM	Support Vector Machine
CNN	Convolutional Neural Network
RNN	Recurrent Neural Network
GRU	Gated Recurrent Unit
RUL	Remaining Useful Life
FFT	Fast Fourier Transform
JANET	Just Another NETwork
FLOPs	FLOating-Point Operations
CPU	Central Processing Unit
GPU	Graphics Processing Unit
